# Evaluation of socio-demographic profile and basic risk factors of tuberculosis patients in South 24 Parganas district of West Bengal, India: a hospital-based study

**DOI:** 10.4314/ahs.v23i3.42

**Published:** 2023-09

**Authors:** Sujay Kumar Bhunia, Sananda Dey, Amitava Pal, Biplab Giri

**Affiliations:** 1 Department of Physiology, University of Gour Banga, Malda 732103, India; 2 Department of Physiology, City College, University of Calcutta, Kolkata 700009, India

**Keywords:** *Mycobacterium tuberculosis*, socio-demographic factors, multi-drug resistance, pulmonary tuberculosis, extra-pulmonary tuberculosis

## Abstract

**Aim:**

To study and analyse the socio-demographic profile and basic risk factors of tuberculosis(TB) patients and their relation with the current epidemiological status of TB registered under the RNTEP program in the study area.

**Subjects and Methods:**

This prospective study was conducted on 1743 newly registered tuberculosis patients at TB-DOT center of South 24 Parganas, West Bengal, India from 2011-2014. Socio-demographic variables and baseline characteristics of the participants were noted by a semi-structured questionnaire.

**Results:**

Our study results indicate that more than 95% of the TB patients were from lower socioeconomic class, and had poor literacy status and tuberculosis was observed highest in non-agricultural labour and cultivators. Among the young adult's majority of the affected population were females from the lower/upper-lower socioeconomic class. Our analysis revealed that, in successful tuberculosis therapy, men were more defaulters than women.

**Conclusion:**

Our study provides a socioeconomic profile and the risk factors of tuberculosis in patients such as the status of therapeutic intervention, involvement of other chronic diseases, age, sex and malnutrition. The findings of this study can be used to plan future studies with specific risk factors of the region and also for implementing the intervention and evaluating its effectiveness.

## Introduction

Tuberculosis (TB) is an ancient chronic infectious disease, recognized as one of the deadliest infectious man-slayers, caused by *Mycobacterium tuberculosis* (MTB). TB development is believed to be influenced by the host and environment-related factors. 1 Although best diagnostic techniques and almost 100% curative treatment regimens for tuberculosis are available nowadays, tuberculosis disease still contributes a major role to the morbidity and mortality in common people of developing countries and affects all age groups.^2^ In TB lung is the most commonly affected organ but it can also affect other organs such as the brain, spine, or kidneys.^3^-^8^ In most cases, TB is curable, however, TB patient can die if he doesn't get proper treatment. According to the WHO report (2020, 2021) globally 7.0 million newly diagnosed people were affected with TB in 2018, 7.1 million in 2019, and 5.8 million in 2020. In 2019, deaths among HIV negative patients and HIV positive patients were 1.2 million and 0.209 million respectively which increased to 1.3 million and 0.214 million respectively in 2020 worldwide.^9^-^10^ In 2020, there was a steep fall on number of newly diagnosed TB patients but the fatality of already diagnosed TB patients were high.^11^ Tuberculosis is a major problem in India, and Southeast Asia accounts for one-third of global TB cases. It was projected that in India, 40% of the population may carry M. tuberculosis (MTB) infection.^12^ Internationally, India ranks as one of the uppermost 5 countries with a high incidence of tuberculosis. 13 In India, the number of occurrences of TB patients (both new and relapse cases) was reported to be 2,4 million, 1.9 million and 1.6 million in 2019, 2020 and 2021 respectively. However, the mortalities reported in Global TB report of 2021 were 0.49 million and 0.08 million in 2020 and 2019 respectively which did not take into account the HIV infected patients. 14-16 , Though, recent scenario has posed COVID-19 as serious life threatening disease, we must not forget the consequences caused by TB .^17^ The World Health Organization (WHO) has planned a TB elimination program set to be implemented by 2030 globally.^18^

The fact that two out of every five Indians are infected with *Mycobacterium tuberculosis*. But, not all of them are developing the disease. Once infected there is a 10% lifetime risk of developing the disease.^4^ TB prevalence, pattern, and death rates vary between regions of a country and between countries. 19 Worldwide socio-demographic factors like age, sex, education, income, lifestyles, etc. play a significant role in the etiology, prevalence, and epidemiological situation of TB. Some social factors include poor life quality, overcrowding, undernutrition, lack of education, lack of awareness, etc. all are inter-related and influence the appearance of TB.^20^-^21^ Several studies indicated an augmented TB incident in patients with immune suppression (HIV/AIDS), smoking, alcohol abuse, and diabetes mellitus.^22^-^25^ The problems of HIV/AIDS, MDR-TB, and adverse tuberculosis treatment outcomes have come up as additional challenges for tuberculosis control.^26^

The national tuberculosis control program (NTCP) in India was started in the year 1962 and presently it is renamed as national tuberculosis elimination program (NTEP). Following a review of NTEP, the Government of India implemented a new regimen, directly observed treatment short-course (DOTS) strategy, and launched revised national TB elimination programs (RNTEP). 4-6,26-27 With the aid of the DOTS strategy recommended by WHO, and RNTEP were successful in bringing down the morbidity and mortality of TB. Under DOTS, socio-economic characteristics are recognized as a decisive role in the success of TB treatment.^28^ The end tuberculosis strategy was started with the goal of TB liberated world and the objective of tuberculosis disposal by 2035. In 2017, the Government of India changed its treatment strategy from intermittent DOTS regimen) to daily DOTS treatment.^29^ The DOTS strategy comprises of two important components viz. systemic monitoring and accountability. This implies that at different levels of the health systems, a systemic recording, reporting, and evaluation of the treatment outcome of all patients are required.^30^

Data on socio-economic characteristics for pulmonary tuberculosis (PTB) and extra-pulmonary tuberculosis (EPTB) are rare. In India, limited studies have been executed to determine the impact of socio-economic status on tuberculosis. Study of case controls, an analytical approach in epidemiology is an important method of detecting the extent to which risk factors are associated with diseases. In the current work, we have taken into account, the socio-demographic contour and basic risk issues of TB patients and their relation with the current epidemiological status of TB registered under the RNTEP program in the area concerned.

## Methods

### Subjects and the study area

This prospective study was conducted on newly registered tuberculosis patients at DOT centers of the tuberculosis unit of Sree Ramakishna Rural Hospital, South 24 Parganas, West Bengal, India from 2011-2014. The study included 1743 patients aged 18 years or more with a confirmed diagnosis of tuberculosis by bacteriologically (smear and culture positive report) at different designated microscopy centers under the tuberculosis unit of Sree Ramakishna Rural Hospital. The research procedure was orally clarified to the participants before data collection to ensure their comprehension and cooperation and informed consent was obtained from them. This study received ethical approval (Ref. No. UGB/IEC (Human)/0013-21, dt. 25.11.2021) and it was carried out in compliance with the Helsinki declaration and the committee's ethical standards.

### Study variables

A semi-structured questionnaire was created after reviewing similar types of literature to record baseline characteristics and socio-demographic variables of the participants such as sex, education, occupation, economic status, treatment status, outcome status, chronic diseases and co-infection, and so on. 31-32 The socio-economic profile of participants was estimated using modified Kuppuswami scale according to Kumar et al. (2013). 33 The participants' height and body weight were measured using standard techniques and landmarks. According to WHO's suggested cut-off values for BMI, the participants were divided into various nutritional groups.

### Statistical analysis

For categorical data, frequencies and percentages were used, while for continuous data, mean and standard deviations were used. The Chi-square test was used to compare categorical qualitative variables between the groups. All the analyses were performed using the Statistical Package for Social Sciences (Version 20).

## Results

**Figure 1:**
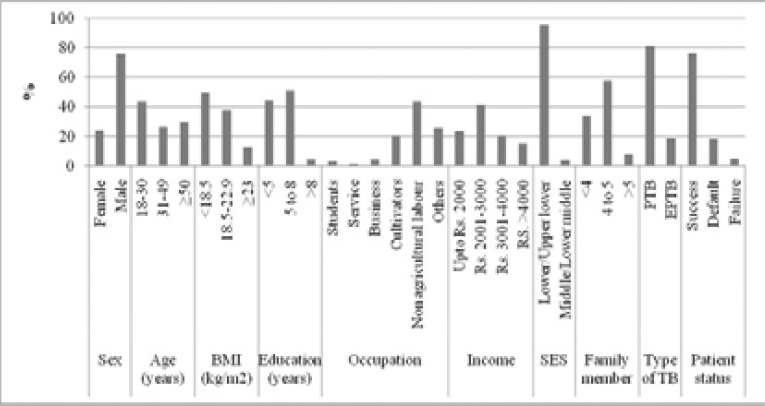
Different socio-demographic and physical characteristics of the tuberculosis patients

As per the selection criteria, 1743 tuberculosis patients were screened. Among the tuberculosis patients, 1324 (75.96%) are male. Table 1 shows the physical characteristics of tuberculosis patients. The average ages of male and female tuberculosis patients were 40.00±16.94 years and 35.80±15.57 years respectively. The average BMI of male tuberculosis patients was 19.22±3.60 kg/m^2^ which was within the normal range. However, the average BMI of female tuberculosis patients was significantly lower than their counterparts; their BMI fell under the underweight category (Fig.1).

**Table 1:** Physical characteristics of the tuberculosis patients

	Male (n = 1324)		Female (n = 419)	
	Mean±SD	Range	Mean±SD	Range
Age (years)	40 ±16.94	18-95	35.8 ±15.57	18-80
Weight (kg)	45.29 ±7.7	25-89	39.86 ±9.2	20-81
Height (cm)	153.86 ±6.55	130-174	152.03 ±5.82	132-171
BMI (kg/m^2^)	19.22 ±3.6	8.96-41.19	17.29 ±4.01	8.22-29.43

Among the tuberculosis patients, around 44% of the participants were aged 30 years or lower. Whereas, about 29% of the participants were aged 50 and up. A large proportion of tuberculosis patients (49.45%) had a BMI of less than 18.5 kg/m2. More than 95 percent of the tuberculosis patients had less than eight years of schooling. In the present study, tuberculosis was observed highest in non-agricultural labour (43.66%) and in cultivators (20.6%). Our present study showed that tuberculosis was most communal in patients who have a family income of less than Rs. 3,001 (64.78%) than patients who have income more than Rs.4,000 (15.26%). More than 95 percent of the tuberculosis patients belonged to were from lower socioeconomic classes. There were 1416 (81.24%) having pulmonary tuberculosis while 45 (2.58%) were diagnosed with multi-drug resistant tuberculosis. Among the 1743 tuberculosis patients, 1331 (76.36%) had completed tuberculosis therapy. Whereas, failure of tuberculosis therapy was 92 (5.28%). The present study showed that 94 (5.39%) patients died of tuberculosis (Table 2; Fig.1).

**Table 2:** Socio-demographic characteristics of the tuberculosis patients (n = 1743)

Variables		f	%	P
Sex	Female	419	24.04	0.000
	Male	1324	75.96	
Age (years)	18-30	760	43.60	0.000
	31-49	466	26.74	
	=50	514	29.49	
BMI (kg/m^2^)	<18.5	862	49.45	0.000
	18.5-22.9	659	37.81	
	=23	222	12.74	
Education (years)	<5	772	44.29	0.000
	5-8	893	51.23	
	>8	78	4.48	
Occupation	Students	55	3.16	0.000
	Service	30	1.72	
	Business	81	4.65	
	Cultivators	359	20.60	
	Non agricultural labour	761	43.66	
	Others	457	26.22	
Income	Upto Rs. 2000	408	23.41	0.000
	Rs. 2001-3000	721	41.37	
	Rs. 3001-4000	348	19.97	
	RS. >4000	266	15.26	
SES	Lower/Upper lower	1660	95.24	0.000
	Middle/Lower middle	73	4.19	
Family member	<4	599	34.37	0.000
	4-5	1002	57.49	
	>5	142	8.15	
Chronic diseases (hypertension, diabetes)		107	6.14	
Type of TB	PTB	1416	81.24	0.000
	EPTB	327	18.76	
Patient status	Success	1331	76.36	0.000
	Default	320	18.36	
	Failure	92	5.28	
Death		94	5.39	
MDR		45	2.58	

The sex variation of tuberculosis and its association with different socioeconomic factors of the study participants was noted and the results were presented in table 3. In comparison to the age groups, tuberculosis was observed lower among female participants (OR: 0.57; CI: 0.44-0.75) with age 50 years or more than male. Similarly, when compared with nutritional status of the participants, it was noted that undernourished (BMI <18.5 kg/m^2^) female (OR: 3.04; CI: 2.34-3.94) were more infected by tuberculosis than male. Around 94 percent of the male patients were from lower/upper-lower socioeconomic class. Whereas, all the female tuberculosis patients belonged to the same socioeconomic class. The outcomes of the present study also revealed the incidence of pulmonary tuberculosis was lower in female patients (OR: 0.47; CI: 0.36-0.60) than that in male. In terms of success in tuberculosis therapy, female (OR: 2.12; CI: 1.57-2.85) were more successfully completed tuberculosis therapy than male. Similarly, female were less defaulters (OR: 0.39; CI: 0.28-0.56) than male.

**Table 3:** Sex variation of socio-demographic characteristics of the tuberculosis patients

Variables		Male (n = 1324)	Female (n = 419)	Chi-Square	OR (95^th^ CI)	p
Age (years)	18-30	542 (40.94)	218 (52.03)	17.729 (0.000)	1	
	31-49	362 (27.34)	104 (24.82)		0.71 (0.55-0.93)	0.014
	=50	420 (31.72)	97 (23.15)		0.57 (0.44-0.75)	0.000
BMI (kg/m^2^)	<18.5	575 (43.43)	287 (68.5)	82.962 (0.000)	3.04 (2.34-3.94)	0.000
	18.5-22.9	566 (42.75)	93 (22.2)		1	
	=23	183 (13.82)	39 (9.31)		1.3 (0.86-1.95)	0.213
Education (years)	<5	590 (44.56)	182 (43.44)	4.27 (0.118)	1.7 (0.9-3.21)	0.104
	5-8	668 (50.45)	225 (53.7)		1.85 (0.98-3.49)	0.056
	>8	66 (4.98)	12 (2.86)		1	
Income	Upto Rs. 2000	313 (23.64)	95 (22.67)	2.438 (0.487)	0.79 (0.55-1.12)	0.185
	Rs. 2001-3000	551 (41.62)	170 (40.57)		0.8 (0.58-1.1)	0.171
	Rs. 3001-4000	268 (20.24)	80 (19.09)		0.77 (0.54-1.12)	0.172
	RS. >4000	192 (14.5)	74 (17.66)		1	
SES	Lower/Upper lower	1251 (94.49)	419 (100.00)	41.143 (0.000)	-	
	Middle/Lower middle	73 (5.51)	-		-	
Family member	<4	768 (58.01)	153 (36.52)	1.17 (0.557)	0.95 (0.63-1.45)	0.829
	4-5	446 (33.69)	234 (55.85)		1.13 (0.89-1.42)	0.322
	>5	110 (8.31)	32 (7.64)		1	
Chronic diseases (hypertension, diabetes)		91 (6.87)	16 (3.82)	5.685 (0.017)	0.54 (0.31-0.93)	0.025
Type of TB	PTB	1116 (84.29)	300 (71.6)	31.353 (0.000)	0.47 (0.36-0.61)	0.000
	EPTB	208 (15.71)	119 (28.4)		1	
Patient status	Success	973 (73.49)	358 (85.55)	27.204 (0.000)	2.12 (1.57-2.85)	0.000
	Default	280 (21.15)	40 (9.55)	32.004 (0.000)	0.39 (0.28-0.56)	0.000
	Failure	71 (5.36)	21 (5.01)	0.08 (0.778)	0.93 (0.56-1.53)	0.78
Death		74 (5.59)	20 (4.77)	0.426 (0.514)	0.85 (0.51-1.4)	0.52
MDR		40 (3.02)	5 (1.19)	4.965 (0.026)	0.39 (0.15-0.99)	0.047

## Discussion

This study is carried out on patients enrolled in diverse DOTS centers of the defined RNTCP areas who are undergoing a combination therapy for the management of tuberculosis in the DOTS program. Socio-economic status and allied risk factors play a crucial role in successful therapy. Early diagnosis and prompt treatment are the keys factors in controlling TB. In the present study, persons suffering from TB had increased odds of decreasing SES (Socio-economic status) for all the studied SES variables (viz. education, occupation, income, family members) studied on univariate analysis. An insight into the socio-economic dimensions of the disease in a community is highly essential. TB is a disease interrelated with poverty, poor life quality, overcrowding, undernutrition, lack of education, lack of awareness, etc. associated with resource-poor countries. 33-34

In our study, we found a strong association between disease progression with poor literacy status whereas, higher education and awareness protect against TB significantly. In this study, non-agricultural labour was one of the key measures of socio-economic status. This study's results indicate that more than 95% of the tuberculosis patients belonged to the lower socio-economic profile and tuberculosis was observed highest in non-agricultural labour (43.66%) and cultivators (20.6%). We found population, the rate of adverse events was higher (41.37%) in the low-income range group (i.e., Rs. 2001-3000) patients followed by the group of income at Rs. 3001-4000 and Rs. > 4000. The adverse events percentage was found highest in the patients group educated in high school (51.23%) and primary school (44.29%).

We observed that male patients are more infected than female patients whose primary occupation was non-agricultural labour. The occurrence of the detected adverse events was found to be maximum in non-agricultural labour (43.66%), and further events were displayed in the others (Housewife, Housework, Retired person) (26.22%) and cultivator groups. A probable explanation for this male predominance is that most men are more involved in societal and labour work as compared to the women in most countries, thus endorsing disease transmission. Tuberculosis disease spreading is allied with family or domestic contact with tuberculosis patients. The risk of *Mycobacterium tuberculosis* infection mostly depends on the proximity of connection with an index case. 35-36

Strikingly, we observed population the majority were women (52.03%) and 40.94% were men among the TB affected were young adults (age 18 to 30) belonging to the lower/upper-lower socioeconomic class. This is most likely that women facing inconsistent family income, low social status, and barely aware delays seek a diagnosis. Socio-economic conditions and cultural factors may curtain health care to them. Our analysis revealed that women (85.55%) completed tuberculosis therapy than male patients (73.49%). Whereas, in terms of default in tuberculosis therapy, men (21.15%) were more defaulters than women (9.55%).

Clinical Characteristics of enrolled patients, including pulmonary and extra-pulmonary TB patients, were notified during the same study period. We have collected tuberculosis therapy outcomes of all enrolled tuberculosis patients and also collected the data of Multidrug-resistant tuberculosis patients. Our data can be seen as broadly representative of the socio determinants situation in South 24 Parganas district and possibly our state West Bengal. Our study provides a socio-economic profile like education, occupation, income, family member, etc. of TB patients.

As revealed by our study, there is a necessity to be delicate to other health care needs of tuberculosis-affected patients. Socio-demographic status, poor literacy, and poverty are traditional dangers for tuberculosis, attributed to malnutrition and inadequate medical attention. 36 This study will be helpful for further understanding of socio-economic risk factors associated with the onset and progression of tuberculosis, and assessment of best intervention and effectiveness.
